# Repetitive transcranial magnetic stimulation as add-on the rapyin persistent postural-perceptual dizziness

**DOI:** 10.1016/j.ibneur.2024.10.005

**Published:** 2024-10-29

**Authors:** Yao Jia, Hongbin Wang, Dan Li, Xingli Wu, Jiawen Yang, Weifei Min, Ting Ma, He Huang, Rui Li

**Affiliations:** a Department of Rehabilitation, Shangluo Central Hospital, Shaanxi 726000, PR China; b Department of Neurology, Shaanxi Provincial People’s Hospital, Xi'an 710061, PR China

**Keywords:** Dizziness, Medication, Persistent postural-perceptual dizziness, Repetitive transcranial magnetic stimulation, Vestibular rehabilitation training

## Abstract

**Background:**

This study aims to evaluate the clinical effectiveness of repetitive transcranial magnetic stimulation (rTMS) when used as an add-on therapy for individuals with persistent postural-perceptual dizziness (PPPD).

**Methods:**

In this randomized controlled, double-blind trial conducted at Shangluo Central Hospital, patients with PPPD diagnosed in the neurology departments were included. Participants were randomized into a rTMS treatment group and a control group in a 1:1 ratio by the randomized grouping method. Patients in both groups received conventional treatment, with the rTMS treatment group underwent daily rTMS sessions, whereas the control group received sham rTMS treatments following the same schedule. The effectiveness of the treatments was primarily assessed using the Dizziness Handicap Inventory (DHI), Hamilton Anxiety Rating Scale (HAMA), and Hamilton Depression Rating Scale (HAMD), which measured symptoms of vertigo, anxiety, and depression at baseline, after two weeks, and at the end of four weeks.

**Findings:**

Of the 46 participants recruited, 2 were excluded due to contraindications, 22 were randomly assigned to the rTMS treatment group, and 22 were assigned to the control group. Ultimately, 2 withdrew for personal reasons, and data from 42 participants were included in the outcome analysis. HAMA, HAMD and DHI scores were significantly lower in the rTMS treatment group than in the control group after 4 weeks of treatment (p<0.05). A positive correlation was also observed between DHI scores and HAMA or HAMD scores.

**Conclusions:**

This pilot study demonstrated that rTMS is a beneficial add-on therapy for patients with PPPD.

## Highlights

• rTMS improves symptoms in PPPD by reducing anxiety and depression.
• rTMS for PPPD patients has minimal side effects.
• rTMS is a beneficial add-on therapy for patients with PPPD.

## 1 Introduction

Persistent postural perceptual dizziness (PPPD) is a chronic functional disorder characterized by balance disorders and dizziness triggered by postural changes or shifts in complex visual environments. The diagnostic criteria for PPPD were established by experts who extensively reviewed research on fearful Phobic postural vertigo (PPV), spatial motion discomfort (SMD), visual vertigo (VV), and were published by the Vestibular Disorders Classification Committee of the Bárány Society(Staab et al., (2017)). Studies have shown that anxiety and depression was associated with PPPD, and these psychological factors tend to intensify as PPPD persists, severely impacting their quality of life(Staab (2020)).Studies have shown that personality traits affect the psychological and spiritual state of patients through genetic, environmental and other factors, and also affect the generation of diseases and the process of diagnosis and treatment(Cerdá et al., (2010)). This study speculated that the personality basis and personality change of PPPD patients affected the perception of dizziness and other symptoms, resulting in dizziness symptoms and fear, anxiety, fear and other emotions in patients, and excessive worry would lead to more serious consequences. Long-term similar experiences led to anxiety, depression and other emotions, which aggravated the dizziness and instability of patients. This leads to prolonged clinical symptoms of PPPD. We excluded patients with mental illness (e.g., depression, generalized anxiety, etc.) before making the PPPD diagnosis. The occurrence of PPPD is closely related to anxiety-related personality traits, and anxiety depression is more common in PPPD patients than in the general population. Studies have shown that personality traits affect the psychological and spiritual state of patients through genetic, environmental, and other factors, and also affect the generation of diseases and the process of diagnosis and treatment. This study speculated that the personality basis and personality change of PPPD patients affected the perception of dizziness and other symptoms, resulting in dizziness symptoms and fear, anxiety, fear and other emotions in patients, and excessive worry would lead to more serious consequences. Long-term similar experiences led to anxiety, depression and other emotions, which aggravated the dizziness and instability of patients. This leads to prolonged clinical symptoms of PPPD.

Our previous study demonstrated that a multi-disciplinary team (MDT) approach tailored for patients experiencing vertigo/dizziness enhances medical management and improves outcomes, including for those with PPPD (Liu et al., (2021)). Currently, PPPD is managed using several methods, including medication, cognitive-behavioral therapy (CBT), vestibular rehabilitation therapy (VRT), and patient education(Staab (2020)). The preferred pharmacological treatments of PPPD are selective serotonin reuptake inhibitors (SSRIs) and serotonin-norepinephrine reuptake inhibitors (SNRIs). However, due to the adverse effects and slow onset of action of these medications, which often require combination therapy, adherence can be problematic, with an overall response rate of 65.0 % for SSRIs in PPPD treatment(Min et al., (2021)). Furthermore, Holmberg et al. (Holmberg et al., (2006)) found that CBT had limited effectiveness in treating PPV, which was not widely adopted in primary care settings, which limited its clinical utility. Vestibular rehabilitation exercises are deemed effective by most PPPD patients, as supported by Thompson et al. (Thompson et al., (2015))Additionally, Nada et al. showed that tailored VRT effectively decreased symptoms and enhanced quality of life in PPPD patients (Nada et al., (2019)). Despite the above interventions reducing symptoms, they have not completely alleviated them (Waterston et al., (2021)), indicating a need for further exploration of more effective treatments for PPPD.

Repetitive transcranial magnetic stimulation (rTMS) is an on-invasive neuro modulation technique that stimulates the cerebral cortex and peripheral nerves, influencing neurotransmitter metabolism and neuro electrical activity in the brain. Studies have demonstrated that rTMS can alleviate symptoms of anxiety and depression (Siddiqui et al., (2023)). The amygdala, insula, anterior cingulate gyrus, prefrontal cortex, and inferior frontal gyrus are the neural cortical areas closely associated with emotions. Dysfunction in these functional areas can disrupt neurotransmitter conduction, which subsequently affects vestibular function and postural reflexes (Trinidade et al., (2020)). Seunghee Na et al. (Avissar et al., (2017)) revealed significant reductions in cerebral blood flow in the insula and frontal regions of patients with PPPD. Moreover, stimulation of the left dorsolateral prefrontal cortex (DLPFC) has been found particularly effective, potentially due to a functional connection between the left DLPFC and the striatum.

Functional magnetic resonance imaging (fMRI) measured activity and connectivity in the vestibular, visual, and anxiety-related areas of the brain(Indovina et al., (2015)), and patients showed reduced stimulus-related activity in the parietal insular vestibular cortex (PIVC), anterior insula, inferior frontal gyrus, hippocampus, and anterior cingulate cortex, which may be related to anxious states. At the same time, the volume of gray matter in the temporal cortex, cingulate cortex, anterior central gyrus, hippocampus, dorsolateral prefrontal cortex, caudate nucleus, and cerebellum decreased. In the visual cortex, auxiliary motor areas, and somatosensory processing structures, the duration of the patient's disease was negatively correlated with the volume of gray matter(Wurthmann et al., (2017)). These results suggest that the functional brain regions responsible for high levels of spatial orientation, multisensory integration, and threat assessment in people with PPPD are not as active or well connected as in normal people, which makes postural and gaze control difficult to integrate effectively at lower levels. The DLPFC is a major brain region that regulates emotions, is a core brain region for executive function, and is capable of allocating attention and/or maintaining task-related information according to task demands, evaluating incoming information as well as internal states to guide appropriate responses to tasks. PPPD symptoms can be aggravated in upright postures, active or passive movements regardless of direction and position, moving visual stimuli, or complex visual environments. We speculate whether rTMS stimulation of DLPFC can be used for neuro regulation. The occurrence of PPPD is closely related to anxiety-related personality characteristics. Compared with the general population, anxiety and depression are more common in PPPD patients, and the left DLPFC is recommended by the guidelines for the treatment of depression(Lefaucheur et al., (2020)). Therefore, this study takes the left DLPFC as the therapeutic target to explore whether rTMS can improve the dizziness symptoms of PPPD.

## 2 Article types

Clinical Trial Article

## 3 Methods

### 3.1 Study design

This study was a double-blind, randomized controlled trial to investigate the efficacy of rTMS as an add-on therapy for PPPD. The study was conducted in Shangluo Central Hospital, a tertiary hospital in western China. It was approved by the Ethics Committee of Shangluo Central Hospital, Ethics Approval No. JS2019058.

### 3.2 Sample size estimation

To detect a between-group mean difference of a 20-point reduction in Dizziness Handicap Inventory (DHI at four weeks, ensuring a power of 80 % at a 2-sided α-level of 0.05 and accounting for an estimated 10 % missing data on the primary outcome), we planned to randomize 44 participants in a 1:1 ratio in this trial. The sample size was calculated using PASS 15 software.

### 3.3 Participants

Eligibility for participation was assessed by participating neurologists who made a clinically definitive diagnosis of PPPD based on the expert consensus issued by the 2017 Bárány Association Vestibular Disorders Classification Committeeet al., 2017). The presence of each of the following five features is required to make the diagnosis: a. The duration of the disease is more than 3 months, manifested by one or more symptoms such as dizziness, instability, or non-rotational vertigo, and the onset of each episode is more than a few hours during most of the month; b. Symptoms are aggravated by factors such as upright posture or active and passive motor or moving visual stimuli and complex visual backgrounds; c. the disorder is triggered by an episode of unsteadiness, dizziness or vertigo - caused by another balance disorder, a neurological or medical disorder, or psychological distress. d. symptoms must cause considerable distress to the sufferer. e. the symptoms should not be better accounted for by an alternative diagnosis (Webster et al., (2023)).We excluded patients with mental illness (e.g., depression, generalized anxiety, etc.) before making the PPPD diagnosis. All participants were required to provide written informed consent.

Patients were not considered for inclusion in the study if, in the opinion of the recruiting neurologist, also exclude individuals suffering from mental illnesses (such as generalized anxiety disorder or major depression), they had a comorbidity of other serious medical conditions that would interfere with their ability to participate in physical therapy. In addition, we excluded the following: patients with metallic implants in the skull, heart, etc; dizziness caused by toxic adverse effects of various medications as well as withdrawal effects; previous history of epilepsy; women who were pregnant or breastfeeding; patients with severe visual and hearing impairments; patients who belonged to patients with contraindications to TMS; those who were not able to participate in 20 rTMS sessions over a period of 4 weeks within 8 weeks; those whose comprehension was insufficient to complete the questionnaires; and those who did not have the ability to consent.

### 3.4 Randomisation and masking

After completing the baseline assessment, participants were randomly assigned by trial staff (1:1) to receive either specialized rTMS treatment in combination with conventional treatment or conventional treatment alone, and random assignment was grouped by a table of random numbers. Treatment allocation was unknown to participants, researchers collecting trial results, and statisticians. Due to the nature of the intervention, it was not possible to blind the trial leader, the rTMS specialist technician.

### 3.5 Procedures

**Fig. 1:**
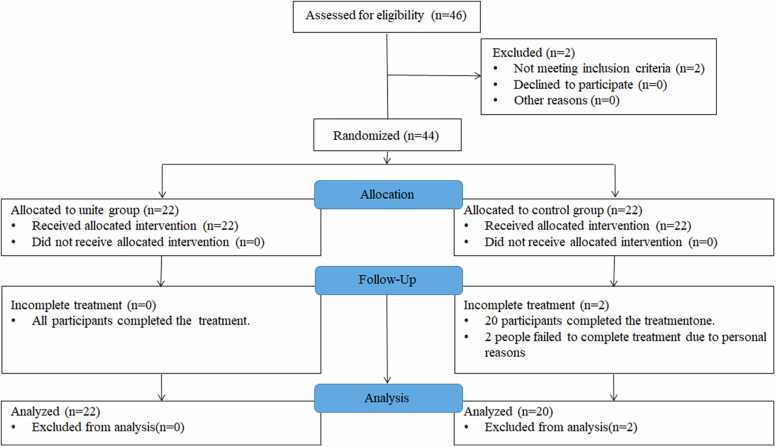
Flow diagram for study participants.

Prior to random assignment, researchers conducted face-to-face interviews with patients who agreed to undergo the study, during which eligibility was screened and informed consent was obtained. Forty-four participants were randomly assigned to the rTMS group (n=22) and the control group (n=22) using the random number table method. The rTMS group underwent true rTMS, medication, and VRT, the control group underwent sham rTMS, medication, and VRT. (Fig. 1).

The DHI, including 25 questions to evaluate the severity of subjective symptoms of vertigo/dizziness, is a widely used clinical scale for assessing the symptoms of dizziness/vertigo (Jacobson (1990)). The 24-item Hamilton Depression Scale (HAMD) (Carrozzino et al., (2020)) and the 14-item Hamilton Anxiety Scale (HAMA) (Thompson (2015)) were used to assess levels of anxiety and depression in PPPD patients. Additionally, to monitor any adverse reactions, a self-designed rTMS Adverse Reactions Scale was utilized. Assessments using the DHI, HAMD, and HAMA scales were conducted at the initiation of treatment, two weeks into the treatment, and after four weeks. Adverse reactions were closely tracked and recorded throughout the trial.

#### 3.5.1 Repetitive transcranial magnetic stimulation treatment

The rTMS device used was the YRD CCY-I magnetic field stimulator, manufactured by Wuhan Yiruide Equipment New Technology Co., Ltd. The treatment parameters were set at an80 % stimulation intensity, a frequency of 10 Hz, a stimulation duration of 3 seconds, with a 25-second interval between stimulations (20 min/session). Treatments were administered five times per week for a duration of four weeks. The control group received pseudo-rTMS treatment, wherein the stimulation coil was rotated by 90° to significantly diminish the intensity of the magnetic field reaching the target site thereby rendering the stimulation ineffective (Jansen et al., (2019)). Additionally, the coil was wrapped in a cloth sleeve filled with a sponge in the middle to ensure that the appearance and working sound were the same as those of the rTMS group. rTMS treatment was administered 1 time/day, 5 times/week, for a total of 20 treatments over 4 weeks. The left dorsolateral prefrontal cortex was localized utilizing a positioning cap designed by TMS manufacturers in accordance with the 10–20 System of electrode placement.

#### 3.5.2 Vestibular rehabilitation training

All patients commenced their VRT at the same time as the rTMS treatment. In the rehabilitation training hall of the hospital, we provided a video tutorial to instruct patients on how to perform VRT, a program established for dizziness patients in our department for the past eight years. At the start of the VRT, the doctor selected an appropriate training menu based on the patient's condition and progressively adjusted the program until the patient was able to complete a full set of training. The doctor guided the entire process to eliminate the interference of different VRT. Patients underwent VRT once a day, completing one full set of training each session, five times a week, over a four-week course. The doctor documented the VRT time and situation of patients on a daily basis and reminded them to persevere in the treatment.

#### 3.5.3 Medications

Anti-anxiety therapy was initiated before the commencement of the study, and to minimize possible side effects, patients were advised to continue maintenance therapy with SSRIs or SNRIs for at least 3 weeks during the study period. The medications used by the participants including Escitalopram (10–20 mg, once a day), Sertraline (50–100 mg, once a day), Fluoxetine (20–60 mg, once a day), and Venlafaxine (75–225 mg, once a day). The specifics of these medications are detailed in Table 1. Statistical analysis showed no statistical difference in the types of drugs taken by the two groups.

**Table 1:** Baseline demographic and clinical characteristics of the study participants.

		Groups (percent)			
Projects		rTMS (n=22)	Control (n=20)	χ2/t	*P*
Age (years)		51.0(15.3)	52.6(13.6)	0.338	0.631
Disease duration (months)		32.2(33.7)	23.6(18.3)	−0.937	0.178
Gender(male/female)		5/17	6/14	0.238	0.625
Precipitating illnesses	BPPV	8(36.4)	7(35.0)		
	VN	5(22.7)	3(15.0)		
	VP	2(9.1)	3(15.0)		
	VM	5(22.7)	3(15.0)		
	Brain injury	1(4.5)	2(10.0)		
	others	1(4.5)	2(10.0)		
	No clear cause				
Medication	Escitalopram	9(40.9)	8(36.4)		
	Sertraline	7(31.8)	6(27.3)		
	Fluoxertine	1(4.5)	2(9.1)		
	Venlafaxine	2(9.1)	3(13.6)		
	With no medications	3(13.6)	3(13.6)		
VRT		20(90.1)	18(90.0)		

### 3.6 Clinical assessment

Patients were assessed by two experienced and professionally trained physicians prior to treatment, at 2 weeks post-treatment, and at 4 weeks post-treatment, respectively. The ultimate outcome of the assessment was the average of the assessment scores of the two doctors, and neither of the doctors was aware of the grouping. (1) Main outcome indicator: DHI. (2) Secondary outcome indicators: HAMA and HAMD.

### 3.7 Statistical analysis

The data were analyzed using SPSS 22.0 software. Independent sample t-tests were used to compare measurement data across groups, and paired t-tests were used to for within-group comparisons. The data normally distributed were expressed as mean (standard deviation). Prior to doing one-way ANOVA, the data were tested for normality and homogeneity of variances using the Lilliefors (Lillietest) and Levene's (Vartestn) tests, respectively. Logistic regression analysis was performed to assess the impact of HAMA and HAMD scores on DHI scores, treating HAMA and HAMD scores as independent factors and DHI scores as the dependent variable.

## 4 Results

### 4.1 Clinical baseline of the participants

Forty-two participants were included in the final analysis, with two individuals from the control group withdrawing from the study due to personal reasons. There were no significant differences in baseline clinical and demographic characteristics between the rTMS group and the control group (p>0.05). The analysis covered precipitating illnesses, clinical status, and medication use among PPPD patients (Table 1).

Gender is the count data, expressed as frequency. Non-continuous variables are presented as numbers and percentages. BPPV, benign paroxysmal positional vertigo; VN, vestibular neuritis; VP, vestibular paroxysm; VM, vestibular migraine. VRT, vestibular rehabilitation therapy.

### 4.2 Clinical outcomes

Prior to therapy, there was no discernible difference in the in the HAMA, HAMD, or DHI scores between the two groups (P=0.161, P=0.047, and P=0.162, respectively). After two weeks of treatment, the scores for DHI, HAMD, and HAMA in the rTMS group showed significant improvement compared to the control group (P=0.047, P=0.003, and P=0.001, respectively). By the fourth week of treatment, these differences had become even more pronounced (P<0.001 for all) (Table 2).

**Table 2:** Summary of clinical variables across time and group.

Measures	Groups [Mean(SD)]		t	*P*
	rTMS (n=22)	Control (n=20)		
HAMD				
0-week	24.09 (12.04)	27.80 (8.89)	−1.10	0.16
2-week	14.60 (9.21)	19.71 (8.90)	−1.81	0.05
4-week	6.09(5.89)	18.35 (9.44)	−4.77	0.00
HAMA				
0-week	23.00 (11.08)	30.00 (11.51)	−1.71	0.06
2-week	13.90 (7.71)	23.45 (12.18)	−2.99	0.00
4-week	7.50 (4.84)	23.95 (13.27)	−4.87	0.00
DHI				
0-week	50.73(27.77)	55.50 (17.88)	−0.99	0.16
2-week	28.89(20.25)	49.10 (20.24)	−3.21	0.00
4-week	11.27 (9.53)	44.60 (19.10)	−6.94	0.00

DHI: dizziness handicap inventory; HAMA: Hamilton anxiety Inventory; HAMD: Hamilton depression Inventory.

Data are expressed as deviations from the mean (SD). Statistical tests were derived from a linear mixed effects model with each clinical variable as the dependent variable and time and treatment (rTMS group vs. control group) as predictors.

The HAMD, HAMA, and DHI dimensions scores for the rTMS group and control groups at weeks 0 and 4 are listed in Table 3. There was no significant difference in the scores of each dimension between the two groups at week 0. At week 0, the control group's mean HAMD and HAMA scores were 27.80 (SD 8.89) and 30.00 (SD 11.51), respectively, while the rTMS group's mean scores were 24.09 (SD 12.04) and 23.00 (SD 11.08). After 4 weeks, the mean HAMD and HAMA scores in the rTMS group fell to 6.09 (SD 5.89) and 7.50 (SD 4.84), and to 18.35 (SD 9.44) and 23.95 (SD 13.27) in the control group. When comparing the scores of the two groups at week four, it was found that the efficacy of the rTMS-treated group was superior to that of the control group. The DHI scores of all dimensions decreased in both the rTMS-treated and control groups when compared with the pre-treatment scores: the total DHI score (11.27±9.53 vs. 44.60±19.10), DHI-P (3.27±3.47 vs. 14.10±6.24), DHI-E (3.45±3.50 vs. 14.10±7.00), and DHI-F (4.73±3.83 vs. 16.70±7.88).

**Table 3:** Intergroup comparison of the rTMS group and control groups [Mean(SD)].

	Groups [Mean(SD)]							
	Control (n=20)		rTMS (n=22)					
	0-week	4-week	0-week	4-week	t	*P*1	f	*P*2
HAMD	27.80 (8.89)	18.35 (9.44)	24.09 (12.04)	6.09 (5.89)	−1.14	0.27	25.99	0.00
HAMA	30.00 (11.51)	23.95 (13.27)	23.00 (11.08)	7.50 (4.84)	−2.01	0.05	29.55	0.00
DHI	55.50 (17.88)	44.60 (19.10)	50.73 (27.77)	11.27 (9.53)	−0.67	0.51	52.67	0.00
DHI-P	17.00 (5.49)	14.10 (6.24)	14.55 (7.84)	3.27 (3.47)	−1.18	0.24	49.51	0.00
DHI-E	18.70 (7.15)	14.10 (7.00)	18.00 (10.74)	3.45 (3.50)	−0.25	0.80	39.93	0.00
DHI-F	22.00 (7.68)	16.70 (7.88)	17.55 (11.68)	4.73 (3.83)	−1.47	0.15	40.41	0.00

*P*1, t analyzed the difference between the experimental group and the control group at week 0 using the independent t-test.

*P*2, f analyzed the difference between the control and experimental groups at week 4 using one-way ANOVA.

DHI: dizziness handicap inventory. DHI-F: the functional score, DHI-E: the emotional score, DHI-P: the physical score. HAMA: Hamilton anxiety Inventory; HAMD: Hamilton depression Inventory.

Table 4 displays the impact of HAMD and HAMA scores on DHI scores. HAMA and HAMD scores were used as independent factors and DHI scores as the dependent variable in logistic regression analysis. Following four weeks of treatment, both the rTMS-treated group and the control group had OR values corresponding to HAMA scores (OR=1.09, OR=2.74) and HAMD scores (OR=2.59, OR=1.98) that were greater than 1, indicating a correlation between lower DHI scores and HAMA and HAMD scores.

**Table 4:** Comparison of the impact of HAMA and HAMD scores on DHI scores.

Times	Measures	rTMS (n=22)			Control (n=20)	
		HAMA	HAMD	HAMA		HAMD
0-week	OR	3.41	3.62	3.16		2.10
	95 %CI	0.46–1.99	0.39–2.18	0.33–1.97		0.07–1.42
	P	0.00	0.00	0.01		0.03
2-week	OR	4.21	7.10	4.39		2.87
	95 %CI	0.65–2.22	1.15–2.78	0.64–2.32		0.42–1.69
	P	0.00	0.00	0.00		0.00
4-week	OR	1.08	2.59	2.74		1.98
	95 %CI	−0.69–0.85	0.21–1.69	0.14–1.87		0.05–1.31
	P	0.83	0.02	0.03		0.04

CI, confidence interval; OR, odds ratio.

Correlation analysis using bivariate Pearson's test showed a positive correlation between HAMD、HAMA scores and DHI scores after two weeks of treatment (rTMS group: r=0.65, r=0.75, Control group: r=0.66, r=0.64, P<0.01). After four weeks of treatment, the rTMS group showed no significant correlation between HAMD and DHI scores (r=0.05, P>0.05) (r=0.05, P>0.05), but the control group continued to show a positive correlation between these scores (r=0.50, P<0.05). For HAMA scores, both groups exhibited a positive correlation with DHI scores (rTMS group: r=0.51, P<0.05 and Control group: r=0.47, P<0.05) (Table 5).

**Table 5:** Correlation analysis between HAMD and HAMA scores and DHI scores.

	0-week DHI		2-week DHI		4-week DHI	
	rTMS group	Control group	rTMS group	Control group	rTMS group	Control group
HAMD						
0-week	0.60**	0.57*				
2-week			0.65**	0.66**		
4-week					0.05	0.50*
HAMA						
0-week	0.56**	0.48*				
2-week			0.75**	0.64**		
4-week					0.51*	0.47*

*and**mean significant correlation at P＜0.05 and extremely significant correlation at P＜0.01.

DHI: dizziness handicap inventory; HAMA: Hamilton anxiety Inventory; HAMD: Hamilton depression Inventory.

Throughout the treatment period, no significant adverse events were reported. Among all patients who completed the treatment, two in the rTMS group experienced mild headaches and discomfort at the site of scalp irritation, while two in the control group reported minor headaches. These symptoms resolved on their own within hours and did not require any intervention, with no statistically significant differences observed between the groups (p>0.05) (Table 6).

**Table 6:** Reported side effects between treatment groups.

Side effects	Treatment group (n=22)	Control group (n=22)	*P*	$\chi^{2}$
Headaches (n)	0	2	0.15	2.10
Scalp irritation (n)	2	0	0.19	1.75
Pain at the stimulation site (n)	0	0	/	/
Sleepiness (n)	0	0	/	/

## 5 Discussion

To date, SSRIs and SNRIs, VRT and CBT or their combination have been proved to be useful to the treatment for PPPD, but all of them had limitations(Miwa and Kanemaru (2022)).This randomized, double-blind, sham-controlled trial found that rTMS, when used as an add-on therapy, significantly improved dizziness symptoms, which was partly contributed to the alleviation of anxiety and depression symptoms in PPPD patients. rTMS was well-tolerated among participants. Our study provided clinical evidence that rTMS may offer further improvement for PPPD.

A meta-analysis suggests that high-frequency rTMS is more effective than low-frequency stimulation for treating anxiety disorders(Cirillo et al., (2019)). Given that patients with persistent postural-perceptual dizziness (PPPD) are often accompanied by anxiety and depression, this study utilized high-frequency and intensity rTMS parameters targeting the left dorsolateral prefrontal cortex (DLPFC), based on previous literatures (Lefaucheur et al., (2020)).

Functional neuroimaging studies in PPPD have reported that the local activity and connectivity in the multimodal vestibular area were decreased and the connectivity between the prefrontal and primary visual areas was increased, which was related to an over-reliance on visual stimuli (Indovina et al., (2021)). Using SPECT imaging, Na, S et al. revealed that rCBF in the frontal, cerebellar and insular cortex of PPPD patients differed from that of healthy individuals, potentially contributing to the impaired read aptation process seen in these patients (Na et al., (2019)). PPPD Patients may have increased sensitivity to interactions between the vestibular, visual, and anxiety systems, which could also heighten the risk of developing anxiety in those with vestibular dysfunction. Mechanisms involved in PPPD development may include the impaired vestibular/visual systems, postural reflexes, and multiple sensory afferents (Indovina et al., (2021)). Clinical guidelines recommend using high-frequency (≈10 Hz) rTMS to stimulate the left DLPFC for depression (Siddiqui et al., (2023)). It has been reported that stimulating the left DLPFC with high-frequency rTMS can alter excitability in frontal and subcortical structures, modulating action potentials in the local cerebral cortex (Lefaucheur JP,Aleman A,Baeken C,Benninger DH,et al., 2023). Thus, we choseto stimulate the left DLPFC at a frequency of 10 Hz. Expectedly, rTMS have positive effects on chronic dizziness, presumably by regulating cognitive functions related to the postural control strategies, stimulating functionally decreased prefrontal regions (Indovina et al., (2021)), and improving coexisting psychiatric symptoms such as depression or anxiety in PPPD patients (Siddiqui et al., (2023)).

In this study, we found that high-frequency rTMS targeting the DLPFC significantly alleviated the symptoms of PPPD patients. After four weeks of treatment, a comparison within the DHI group revealed that scores across all dimensions of DHI were lower than those before treatment in both groups. Logistic regression analysis showed that, after two and four weeks of treatment, the HAMA scores in both TMS-treated group and the control group corresponded to OR values greater than 1, indicating a correlation between the reduction of DHI scores and the decrease in HAMA and HAMD scores. The findings suggested that the decrease in DHI was partially attributable to the reductions in anxiety and depression, indicating that the therapeutic effects of rTMS may be mediated through the alleviation of these psychiatric symptoms in PPPD patients.

## 6 Limitation

This study has several limitations. Firstly, the sample size was relatively small, which may limit the generalizability of the findings. Second, the duration of the intervention only allowed for an assessment of the short-term effectiveness of transcranial magnetic stimulation in treating PPPD, and the long-term effects of the therapy remain unknown. These findings highlight the need for further research with longer follow-up periods to fully evaluate the sustained impact of this treatment.

## 7 Conclusion

This study is the first to demonstrate that rTMS is an effective adjunct therapy for treating PPPD. It is assumed that rTMS positively impacts chronic vertigo by alleviating depression or anxiety in patients with PPPD (Leung et al., (2020)). The findings from this study contribute valuable data to the body of randomized controlled trials on rTMS for PPPD and underscore the need for further research into the effects of rTMS on PPPD.

## Ethics statement

Ethics approval was obtained from the Ethics Committee of Shangluo Central Hospital, Ethics Approval No. JS2019058. In addition, we have taken the participants’ permission and consent to participate in this study.

## Funding

This work was supported by the Project for Xi'an Municipal Agency of Science and Technology (2023JH-YXYB-0094).

## CRediT authorship contribution statement

**Xingli Wu:** Software, Methodology, Data curation. **Jiawen Yang:** Validation, Software, Resources, Investigation. **Yao Jia:** Writing – original draft, Validation, Software, Investigation, Data curation. **Rui Li:** Validation, Project administration, Methodology, Formal analysis, Conceptualization. **Hongbin Wang:** Visualization, Validation, Project administration, Funding acquisition. **Dan Li:** Resources, Investigation, Formal analysis. **Ting Ma:** Visualization, Validation. **He Huang:** Methodology, Investigation. **Weifei Min:** Resources, Methodology, Investigation.

## Declaration of Competing Interest

The authors declare that they have no known competing financial interests or personal relationships that could have appeared to influence the work reported in this paper.
